# Observation of the effect of posterior scleral reinforcement combined with orthokeratology and 0.01% atropine in the treatment of congenital myopia: a case report

**DOI:** 10.1186/s12886-023-03211-w

**Published:** 2023-11-28

**Authors:** Chunxiao Yan, Fangkun Zhao, Shang Gao, Xiaoyu Liu, Taorui Yu, Yanan Mu, Lijun Zhang, Jun Xu

**Affiliations:** 1 The Third People’s Hospital of Dalian, Dalian Municipal Eye Hospital, Dalian Municipal Cancer Hospital, Liaoning Provincial Key Laboratory of Cornea and Ocular Surface Diseases, Liaoning Provincial Optometry Technology Engineering Research Center, Dalian, Liaoning China; 2 Dalian Medical University, Dalian, Liaoning China; 3 The Fourth Affiliated Hospital of China Medical University, Shenyang, Liaoning China

**Keywords:** Congenital myopia, Posterior scleral reinforcement, Orthokeratology, Atropine, Myopia control

## Abstract

**Background:**

Myopia has recently emerged as a significant threat to global public health. The high and pathological myopia in children and adolescents could result in irreversible damage to eye tissues and severe impairment of visual function without timely control. Posterior scleral reinforcement (PSR) can effectively control the progression of high myopia by limiting posterior scleral expansion, improving retrobulbar vascular perfusion, thereby stabilizing the axial length and refraction of the eye. Moreover, orthokeratology and low concentrations of atropine are also effective in slowing myopia progression.

**Case presentation:**

A female child was diagnosed with binocular congenital myopia and amblyopia at the age of 3 and the patient’s vision had never been rectified with spectacles at the first consultation. The patient’s ophthalmological findings suggested, high refractive error with low best corrected visual acuity, longer axial length beyond the standard level of her age, and fundus examination suggesting posterior scleral staphyloma with weakened hemodynamics of the posterior ciliary artery. Thereby, PSR was performed to improve fundus health and the combination of orthokeratology and 0.01% atropine were performed to control the development of myopia. Following up to 8 years of clinical treatment and observations, the progression of myopia could be well controlled and fundus health was stable.

**Conclusion:**

In this report, 8-year of clinical observation indicated that PSR could improve choroidal thickness and hemodynamic parameters of the retrobulbar vessels, postoperative orthokeratology combined with 0.01% atropine treatment strategy may be a good choice for myopia control effectively.

## Background

The prevalence of myopia is increasing globally, with more than 1.4 billion people affected. Among them, high myopia accounts for approximately 2.7% [1]. According to a survey, the global prevalence of high myopia is projected to reach 9.8% by 2050 [2]. Consequently, visual impairment caused by high myopia is projected to affect approximately 55.7 million people, with 18.5 million at risk of blindness [3]. Recently, there has been a growing trend of myopia occurring at a younger age, leading to a longer course and an increased prevalence of high and pathological myopia [4]. Complications arising from pathological myopia are usually irreversible and can severely impact a patient’s visual function [5]. Congenital myopia commonly manifests in preschool children, accompanied by refractive errors, lengthening of the axial and tesselated fundus, progressive myopia, and irreversible damage [6]. Therefore, early intervention is necessary to prevent the development of high and pathological myopia, which can cause severe damage to visual function. Posterior scleral reinforcement stabilizes the eye’s axis and refraction by attaching a reinforcing material to the weakened posterior sclera. This approach enhances scleral resistance, promotes collagen reconstruction, and limits posterior scleral expansion [7]. Allograft sclera is currently the most commonly used reinforcement material because of its high biocompatibility [8] and ability to improve blood supply to the posterior pole of the sclera [9, 10]. Recently, orthokeratology lenses have effectively controlled and delayed adolescents’ myopia progression [11]. The reverse geometric design of these lenses inhibits the increase of the eye axis and delays myopia development by changing the morphology of the cornea’s anterior surface [12–15], resulting in myopic defocus in the peripheral retina [16]. Atropine has been used for myopia control since the 19th century; however, its precise mechanism of action remains unclear [17]. However, studies have shown that atropine acts as an antagonist to muscarinic receptors, thereby affecting the remodeling of the scleral matrix and inhibiting the growth of the eye axis [18]. The level of myopia control by atropine was thought to be concentration-dependent, however, lower concentrations of atropine could also have myopia control and was safer compared to higher concentrations [19]. In recent years, a number of studies have suggested that 0.01% atropine may be a safe and effective choice for controlling the eye axis length [20–24].

Furthermore, research has shown that combining orthokeratology with atropine improves myopia control compared to orthokeratology alone [11]. Here, we report a case of a 3-year-old child with congenital myopia approaching high myopia. The child underwent posterior scleral reinforcement surgery in both eyes in 2015, resulting in the resolution of amblyopia within the first postoperative year. As of 2023, no complications have been observed during the 8-year postoperative follow-up period, and the patient’s myopic refraction, axis length, corrected visual acuity, and fundus condition have remained stable. The patient is undergoing myopia control treatment, and our team will continue to follow up on the patient’s progress.

## Case presentation

**Fig. 1:**
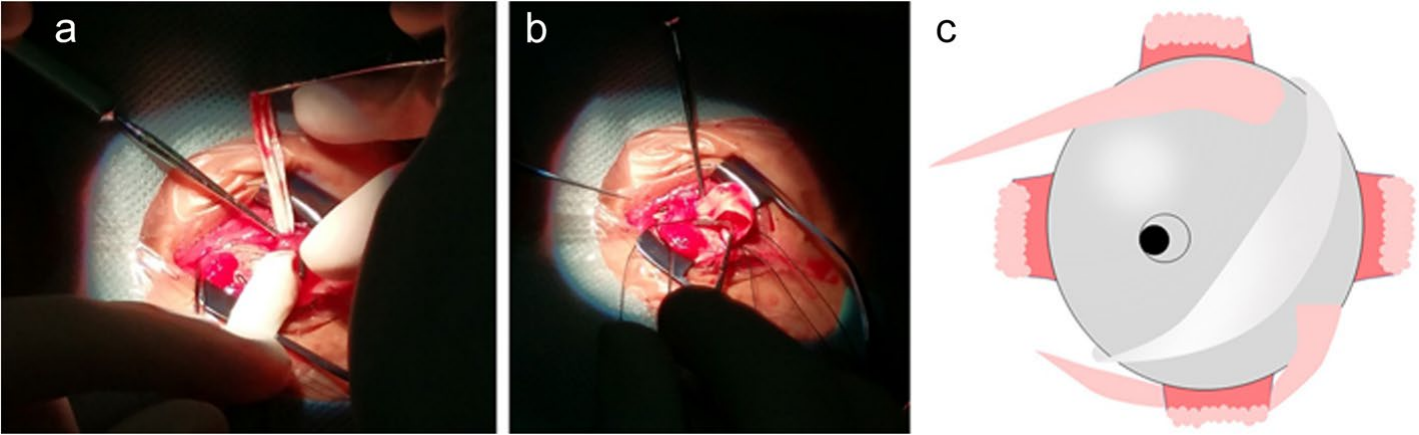
Diagram of PSR of the right eye (“**a**” shows the scleral strip crossing underneath the inferior oblique muscle, “**b**” shows the scleral strip crossing  underneath the external rectus muscle, “**c**”shows the strip wrapping the posterior pole of the eye and the scleral grapevine area in a “U” shape)

**Fig. 2:**
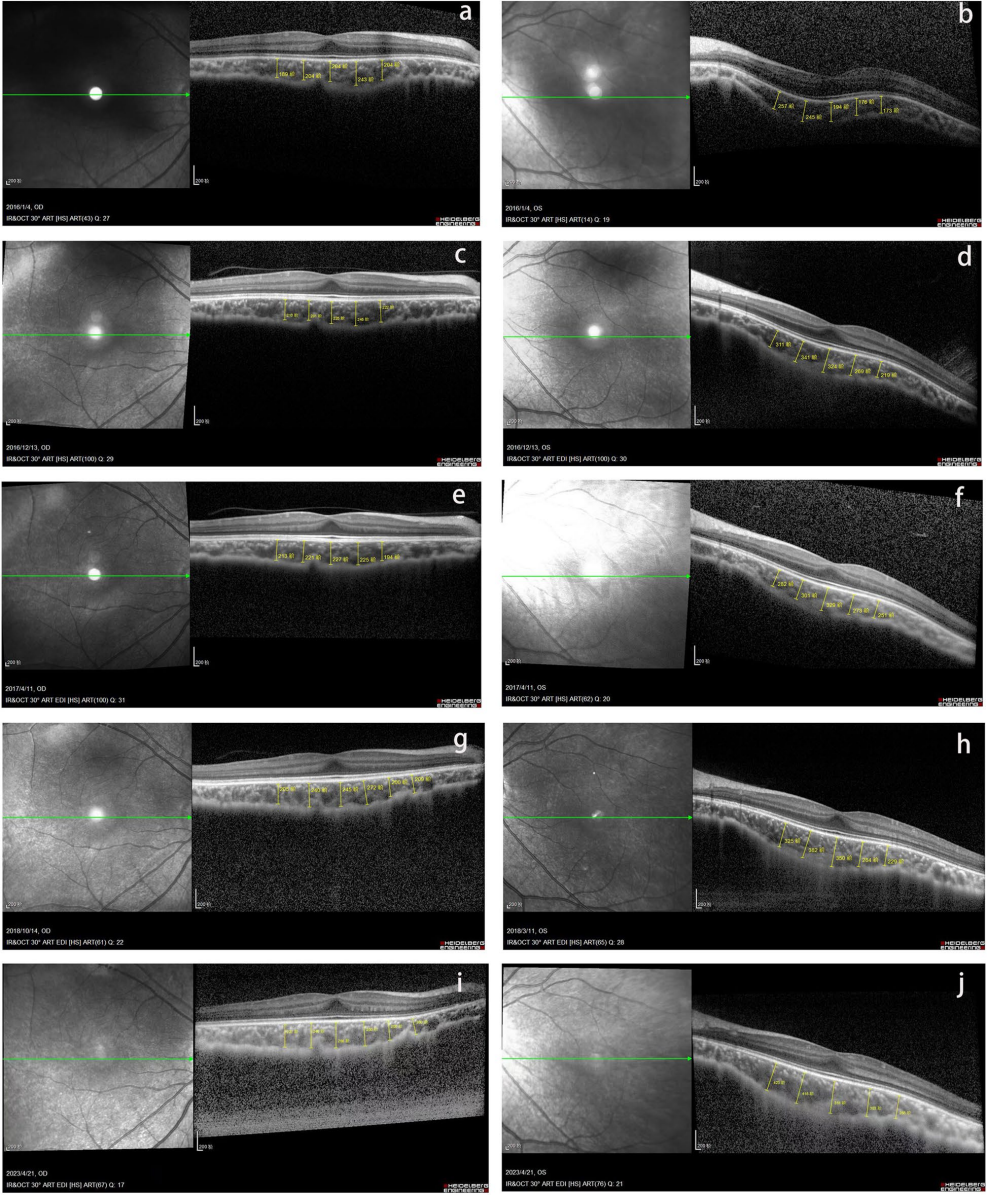
Changes in the subfoveal choroidal thickness (SFCT) of the patient between 2015 and 2023 ("**a**, **b**" are preoperative SFCT, "**c**, **d**" are 1-year postoperative SFCT, "**e**, **f**" are 2-year postoperative SFCT, "**g**, **h**" are 3-year postoperative SFCT, "**i**, **j**" are 8-year postoperative SFCT)

**Fig. 3:**
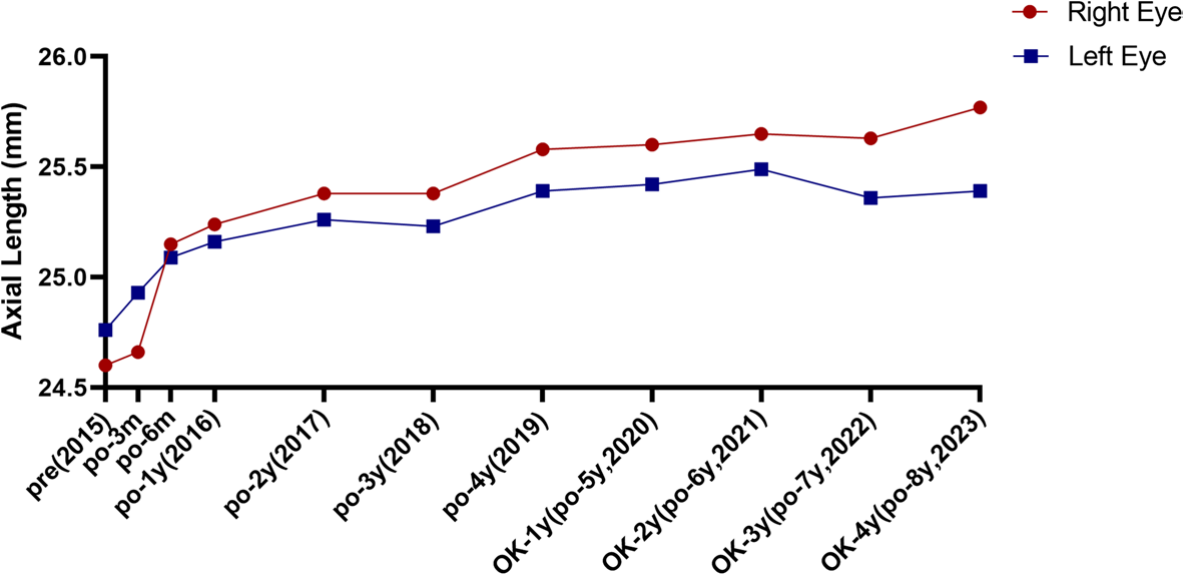
Changes of axial length in patient with bilateral posterior scleral reinforcement combined with orthokeratology and 0.01% atropine from 2015 to 2023 (application of orthokeratology and 0.01% atropine started from 2020 to 2023)

**Fig. 4:**
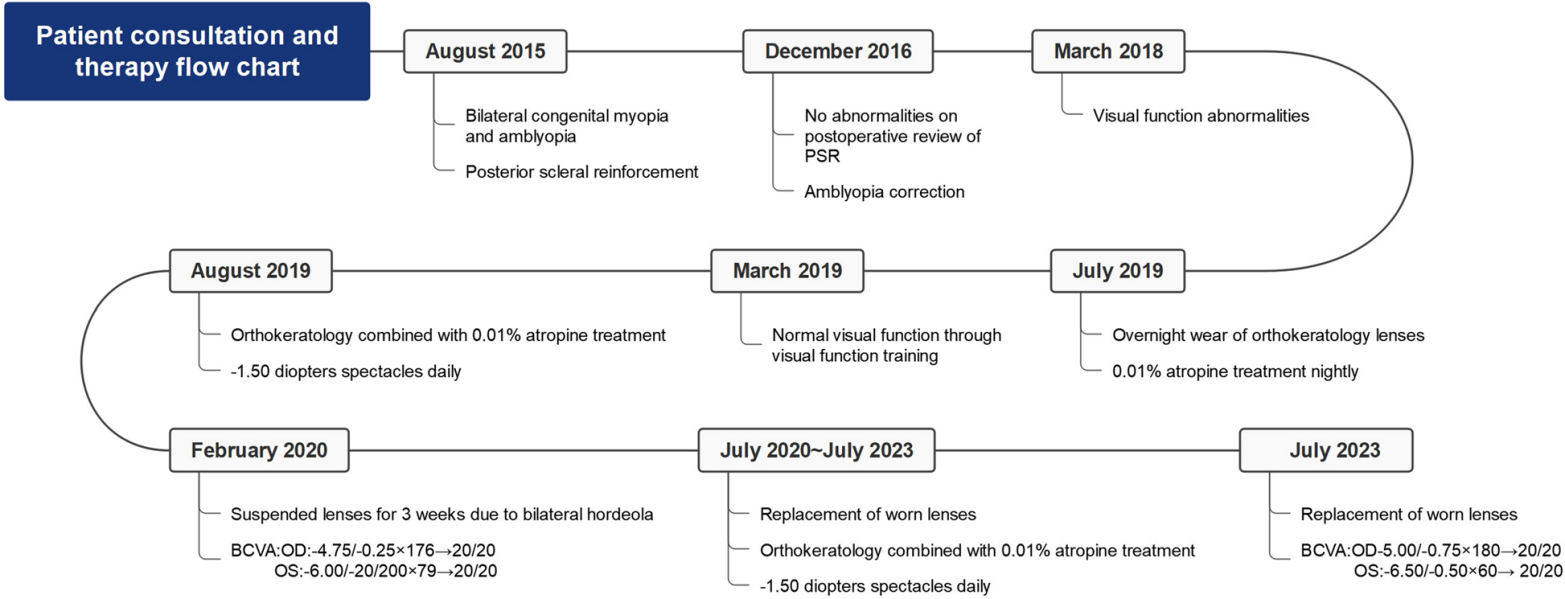
Patient consultation and therapy flow chart

The child was born in March 2012. She was diagnosed at the Refractive Clinic of the Fourth Hospital of China Medical University in August 2015. The parents reported a decline in the child’s visual acuity for 3 months in both eyes and the patient’s vision had never been rectified with spectacles at the first consultation. Uncorrected visual acuity (UCVA) was measured at 20/160 in the right eye and 20/200 in the left during their visit. Objective refraction was − 7.50/-1.00 × 151 diopters in the right eye and − 9.00/-2.00 × 43 diopters in the left. The corrected refraction was − 5.25/-0.75 × 180 diopters in the right eye and − 5.50/-1.00 × 55 diopters in the left after Atropine Sulfate EyeGel to paralyse the ciliary muscle. The best corrected visual acuity (BCVA) was 20/125, and intraocular pressure was 16 mmHg in both eyes. Axial length was 24.60 mm in the right eye and 24.76 mm in the left. Flat meridian and steep meridian were 41.37 D and 43.12 D in the right eye, and flat meridian and steep meridian in the left eye were 42.37 D and 43.75 D. No abnormalities were observed in the anterior segment of both eyes, and the examination of ocular fundus revealed a tessellated fundus and conus. B-mode ultrasonography of both eyes revealed posterior scleral staphyloma and mild vitreous opacities. The sub-foveal choroidal thickness was 235 μm in the right eye and 214 μm in the left. Hemodynamic examination of the retrobulbar vessels in both eyes revealed reduced posterior ciliary artery hemodynamic parameters. The clinical diagnoses were binocular congenital myopia and amblyopia. Considering the patient’s early age of myopia onset, high degree of myopia, significant growth of the eye axis, and the presence of posterior scleral staphyloma in both eyes, we suggested to perform PSR surgery to control expansion of the posterior sclera and improve retrobulbar blood circulation. After excluding contraindications to surgery, posterior scleral consolidation was performed in both eyes under general anesthesia in August 2015. Starting from the inferior temporal area, using the operating microscope, a 270° incision was made in the bulbar conjunctiva and Tenon’s capsule along the outer 2–3 mm of the corneal rim. The sclera was exposed, and the external and inferior rectus muscles were carefully separated. The deep pulling hook was used to assist with exposure, while a small strabismus hook aided in separating the inferior temporal fascia. The top end of the inferior oblique muscle became visible, and the muscle was hooked while keeping it intact. A 60–70 mm long, 6–8 mm wide allograft scleral strip was passed between the inferior oblique muscle and sclera (Fig. 1a). The upper end of the strip passed under the belly of the external rectus muscle (Fig. 1b), while the lower end passed under the belly of the inferior rectus muscle. The strip was then wrapped in a “U” shape around the posterior pole of the eye and scleral staphyloma area (Fig. 1c.). After spreading the strip, it was carefully tightened to abut the scleral wall, ensuring caution to avoid contact with the vortex vein. The free ends are sutured and fixed to the superficial sclera, 6 mm above the temporal and below the nasal area from the corneal rim, respectively (Scleral suture points 5:00–11:00 in the right eye and 1:00–7:00 in the left eye). The bulbar conjunctiva was sutured, and subconjunctival injection of dexamethasone 2.5 mg was administered. Tobramycin-dexamethasone ophthalmic ointment was applied to the conjunctival sac, and the operated eye was bandaged. Postoperatively, the patient was administered anti-inflammatory and anti-infective treatment with systemic dexamethasone injection (2.5 mg) + 250 ml saline intravenous drip for 5 consecutive days, and topical ofloxacin eye drops and 0.1% fluorometholone eye drops for 2 consecutive weeks in both eyes. The patient was discharged on the 6th postoperative day with mild conjunctival congestion in both eyes, preserved corneal transparency, and the fundus status remained unchanged. Visual acuity, axial length, choroidal thickness under the central macular recess, and blood flow status of the retrobulbar vessels were recorded at various time points after postoperative scleral reinforcement, as shown in Table 1. The naked visual acuity improved to 20/100 in both eyes on postoperative day 1 (preoperative VOD:20/160, VOS:20/200), and the corrected visual acuity improved to VOD:20/40, VOS:20/50 on postoperative day 6 (preoperativeVOU:20/125). The corrected visual acuity was 20/20 in the first year after surgery, and the amblyopia resolved. The refractive status of both eyes stabilized within 3 years after surgery. The axial increase in both eyes within 3 years after surgery was 0.26 mm/year in the right eye and 0.16 mm/year in the left. Furthermore, 3 years after surgery, the choroidal thickness under the central macular sulcus was greater than before surgery (Fig. 2). The post-bulbar vascular flow status returned to normal at the 6-month postoperative follow-up, and all post-bulbar vascular flow parameters were normal at the subsequent follow-up. In the 3rd year of postoperative follow-up, the child exhibited poor gaze stability, suppression of the central recess in the left eye, and no stereopsis. Visual function training was provided, and after 1 year, the patient’s perceptual eye position and gaze stability reached the standard and remained stable. In the 4th year after surgery, the patient received binocular orthokeratology (vision shaping treatment design), and 0.01% atropine eye drops to further control myopia progression. The patient’s refraction was − 5.00/-0.75 × 180  diopters in the right eye and − 6.50/-0.50 × 60 diopters in the left eye. Circular corneal shaping lenses were chosen for matching, and 0.01% atropine eye drops were applied to both eyes half an hour before wearing the lenses. Visual acuity, refraction, axial length, and the ocular surface status during the 4 years of treatment with orthokeratology combined with low-concentration atropine are shown in Table 2. At 1 month of orthokeratology, the visual acuity stabilized at 20/25 in both eyes, and the patient accepted the correction of -1.50 diopters, while the BCVA remained 20/20. Patients were instructed to wear frames for daily activities and maintain good distance vision. Atropine was administered once a night at bedtime, one drop at a time. After 8 months, the patient discontinued wearing orthokeratology lenses for 3 weeks because of bilateral hordeola. During this time, the refraction was − 4.75/-0.25 × 176 diopters in the right eye and − 6.00/-1.00 × 79 diopters in the left. The changes in the axial length over the 8 years of patient follow-up (Fig. 3) showed an average growth rate of 0-0.05 mm/year. The ocular surface tear film status was normal, and the flow chart showed the process of patient’s treatment and follow-up (Fig. 4).

**Table 1:** Uncorrected Visual Acuity (UCVA), Refraction & Corrected visual acuity, Axial Length (AL), Subfoveal Choroidal Thickness (SFCT), and Hemodynamic parameters of retrobulbar vessels after Posterior Scleral Reinforcement (PSR)

	UCVA		Refraction & Corrected visual acuity		AL (mm)		SFCT (μm)		Hemodynamic parameters	
	OD	OS	OD	OS	OD	OS	OD	OS	OD	OS
Pre-PSR	20/125	20/125	-5.25/-0.75 × 180→20/160	-5.50/-1.00 × 55→ 20/200	24.60	24.76	204	194	PCA↓	PCA↓
Po-1d	20/100	20/100	/	/	/	/	/	/	/	/
Po-3d	20/100	20/100	/	/	/	/	/	/	/	/
Po-6d	20/100	20/100	-5.00/-0.50 × 180→20/40	-5.50/-0.75 × 55→20/50	/	/	/	/	/	/
Po-1 m	20/200	20/160	-5.00/-0.75 × 180→20/32	-5.50/-0.75 × 55→20/50	/	/	/	/	/	/
Po-3 m	/	/	-5.00/-0.75 × 180→20/32	-6.00/-0.75 × 55→20/40	24.66	24.93			/	/
Po-6 m	/	/	-5.00/-0.75 × 180→20/32	-6.00/-0.75 × 55→20/40	25.15	25.09	/	/	Normal	Normal
Po-1y	/	/	-5.25/-0.75 × 180→20/20	-6.00/-0.75 × 55→20/20	25.24	25.16	225	324	Normal	Normal
Po-2y	/	/	-5.00/-0.75 × 180→20/20	-5.75/-0.50 × 55→20/20	25.38	25.26	227	329	Normal	Normal
Po-3y	/	/	-5.00/-0.75 × 180→20/20	-5.75/-0.50 × 55→20/20	25.38	25.23	245	350	/	/

"/" indicates that the patient has not undergone a follow-up examination

**Table 2:** The effect of orthokeratology combined with 0.01% atropine on refraction & Corrected visual acuity, axial length (AL) and tear film status

	Refraction & Corrected visual acuity		AL (mm)		Tear film	
	OD	OS	OD	OS	OD	OS
Pre-OK	-5.00/-0.75 × 180→20/20	-6.50DS/-0.50 × 60→ 20/20	25.58	25.39	Level 0	Level 0
OK-1d	/	/	/	/		
OK-7d	/	/	/	/		
OK-1 m	-1.50DS→20/20	-1.75DS→ 20/20	/	/	Level 0	Level 0
OK-3 m	-1.50DS→20/20	-1.75DS→ 20/20	/	/	Level 0	Level 0
OK-6 m	-1.50DS →20/20	-1.75DS→ 20/20	/	/	Level 0	Level 0
OK-8 m	-4.75/-0.25 × 176→20/20	-6.00/-20/200 × 79→20/20	/	/		
OK-10 m	-1.50DS→20/20	-1.75DS→ 20/20	/	/	Level 0	Level 0
OK-1y	-1.50DS→20/20	-1.75DS→ 20/20	25.60	25.42	Level 0	Level 0
OK-2y	-1.50DS→20/20	-1.75DS→ 20/20	25.65	25.49	Level 0	Level 0
OK-3y	-1.50DS→20/20	-1.75DS→ 20/20	25.63	25.36	Level 0	Level 0
OK-4y	-5.00/-0.75 × 180→20/20	-6.50/-0.50 × 60→ 20/20	25.77	25.39	Level 0	Level 0

"/" indicates that the patient has not undergone a follow-up examination

## Discussion

Many factors, including environmental and genetic factors, interact to result in myopia [25]. Genetic and embryonic developmental abnormalities mainly cause congenital myopia, manifesting as axial myopia and typical ocular tissue changes [26]. Pathological myopia (PM) refers to a series of degenerative changes in the eye’s fundus because of excessive elongation of the eye axis, with a refraction > -6.0 D or an eye axis > 26.5 mm [27]. In congenital myopia, the excessive growth of the eye axis can easily progress into pathological myopia, causing irreversible visual impairment. Posterior staphyloma (PS), a common fundus change in pathological myopia, is a morphological change in the fundus caused by axial elongation of the eye axis [28], which affects local blood circulation in the macula [29]. Histological studies have revealed a thin sclera, decreased choroidal thickness, and misalignment of scleral collagen fibers at the edge of the uveitic zone [30]. Reinforcing a graft (biological or synthetic material) in the weak sclera at the posterior pole of the eye increases scleral stress and promotes collagen reconstruction, thereby preventing further progression of the eye axis and pathological myopia [8]. In this case, the patient was diagnosed with congenital myopia and amblyopia in both eyes at the age of 3. The fundus examination revealed posterior scleral staphyloma, and the length of the eye axis had already exceeded the level of the adult eye axis (23–24 mm). Considering these factors, posterior scleral consolidation was performed in both eyes after excluding contraindications to surgery. It is hoped that PSR would improve the patient’s fundus health and further have a positive effect on myopia control.

The procedure used the modified Snyder-Thompson approach to transplant the allograft scleral tissue. The modified Snyder-Thompson approach is a widely used procedure that involves creating a U-shaped pocket wrapped around the macular and posterior scleral vitreous areas, allowing for individualized pressure, widening, and padding. It has been demonstrated that posterior scleral reinforcement effectively controls the lengthening of the eye axis, stabilizes the refractive state, and has a low postoperative complication rate [31, 32]. Li et al. [33] observed that the mean eye axis length in the PSR group 5 years after the procedure (29.79 ± 1.26 mm) was significantly shorter than that in the control group (30.78 ± 1.30 mm), effectively controlling the lengthening of the eye axis and myopic progression in pathological myopic eyes. Széll et al. [34] also observed an improvement in BCVA, averaging 0.15 ± 0.09 D in the PSR group 5 years after the procedure, compared to almost no change in refraction in the control group (0.01 ± 0.1 D). In addition, patients with amblyopia showed a more significant improvement in BCVA after undergoing posterior scleral consolidation (0.35 ± 0.12 D). In retrospect, the ocular refractive status of our patient remained stable 4 years postoperatively, with corrected refractions of -5.00/-0.75 × 180 diopters in the right eye and − 6.50DS/-0.50 × 60 diopters in the left. After 1 year postoperatively, both eyes archived a BCVA of 20/20, resulting in the resolution of amblyopia.

Although there was a tendency for the eye axis length to increase within the first 4 years after surgery, the increase was minimal. According to the Public Health Ophthalmology Branch of the Chinese Society of Preventive Medicine [35], the growth rate of the axis length occurs from birth to 3 years of age, with a total growth of approximately 5 mm over that period. From age 3–6 years, the average total growth length of the axis is no more than 1 mm (21.5-22.5 mm), and for Chinese school-age children between 6 and 15 years, the average total growth length of the axis length is 0.93 mm. The growth of the axis length from 6 to 11 years was incremental. The trend of large and rapid growth was 0.09 mm/year for school-age children after 6 years of age, 0.10 mm/year to 0.22 mm/year for 7–8 years of age, 0.13 mm/year to 0.18 mm/year for 9–11 years of age, and 0.01 mm/year to 0.06 mm/year for 12–15 years of age. This patient underwent posterior scleral consolidation in both eyes at the age of 3 years, and the average postoperative axial increase was 0.26 mm/year in the right eye and 0.16 mm/year in the left eye, with the average total length of growth controlled within 1 mm. Posterior scleral consolidation effectively controlled the axis growth in this patient with congenital myopia. It is believed that the improvement in the blood flow status of the retrobulbar vessels and the corresponding change in the axis length because of choroidal thickening contributed to this control. Although conjunctival edema was observed within a short postoperative period, no rejection of the graft material or any other serious and lasting complications, such as elevated intraocular pressure, optic nerve compression, retinal detachment, or retinal hemorrhage, were observed.

The choroid is rich in vascular tissue and essential for maintaining eye physiology. Evidence suggests that the choroid is crucial in controlling eye elongation and developing refractive errors [36, 37]. Animal studies have shown that the choroidal thickness is significantly thinner in models of myopia than in those of hyperopia during choroidal development [38, 39]. The choroidal thickness in the myopia model may be attributed to the short-term thickening of the choroid, which continues to reduce extracellular matrix molecule synthesis and slows eye development [39]. In addition, some clinical studies have suggested the role of choroidal thickness changes in myopia development. Zheng et al. [40] observed an increase in choroidal thickness 1 month after PSR, but there was no statistically significant difference from the control group. Peng et al. [41] found statistically significant differences in choroidal thickness changes between groups from 2 to 3 years after PSR, demonstrating that PSR significantly inhibited eye elongation and induced alterations in choroidal thickness beyond 2 years postoperatively [42]. In this case, the sub-foveal choroidal thickness (SFCT) was found to be thickened from 1 to 2.5 years postoperatively, and ocular ultrasound showed that the hemodynamic parameters of the ophthalmic, central retinal, and posterior ciliary arteries returned to normal 6 months postoperatively. The superficial and deep retinal perfusion in the area increased significantly from the preoperative levels ([33.82 ± 4.33% and 14.29 ± 3.89%] to [48.18 ± 4.56% and 31.47 ± 5.11%]).

Recently, the pathogenesis hypothesis of myopia has suggested that the sclera is the ultimate effector of myopia development, and scleral microenvironmental hypoxia triggers extracellular matrix remodeling and myopia [43, 44]. Conversely, increased choroidal perfusion effectively improves scleral hypoxia and inhibits axis length elongation and myopia development [45]. In this case, the choroidal thickness improved compared to the preoperative level, and amblyopia was cured at 1 year postoperatively. The recovery of visual acuity may be related to the improved blood flow status of the choroid, enhancing visual sensitivity.

In this case report, the patient was found to have a high refractive error with amblyopia during visual development. As children’s physical development progresses, myopia was likely to increase, which could lead to abnormal visual function and binocular gaze stability, fusion, and adjustment, even serious fundus complications. After 3 years of surgery, it was observed that the child exhibited fusion dysfunction. Consequently, the child underwent 1 year of visual function training to stabilize the perceptual eye position, enhance gaze stability and restore normal stereopsis function.

In the 1960s, orthokeratology lenses were first proposed for controlling development [46]. The lens material was upgraded from the early rigid polymethylmethacrylate material to a highly oxygen-permeable fluorosilicone acrylate polymer. A possible mechanism for controlling myopia progression is now attributed to the myopic eye’s relative peripheral refraction, which exhibits myopic defocus with orthokeratology lenses and slows the elongation of the axis length [44, 47, 48]. Recently, with optimized lens design and standardized fitting processes, many clinical trials have shown that orthokeratology lenses can effectively control myopia development with a certain degree of safety [49]. Charm et al. [14] found that high myopia patients wearing orthokeratology lenses could effectively control the axial length growth, with an average increase of 0.19 mm over 2 years, compared to an average increase of 0.51 mm in the control group. Atropine is a competitive muscarinic receptor inhibitor. It is believed that atropine prevents and controls myopia by regulating the ciliary muscles. Although the molecular mechanisms by which atropine controls myopia progression are not fully elucidated, most current studies suggest that atropine may achieve myopia control by acting on relevant receptors and signaling pathways in the retina and posterior sclera. These include M1/M4 receptors in the retina that perform biological functions [50], the cholinergic signaling pathway that mediates retinal M receptors [51], and the G protein signaling pathway that mediates M receptors in the retinal and scleral tissues [52, 53].

Furthermore, atropine has been shown to control the growth of the ocular axis by antagonizing muscarinic receptors and inhibiting fibroblast proliferation in the scleral collagen matrix [18]. In addition, atropine may affect γ-aminobutyric acid, dopamine receptors, and α_2_ adrenergic receptors to control myopia progression. However, further scientific studies and clinical observations are needed, as the involvement of the receptors and signaling pathways is currently at the animal study level. Notably, the effectiveness of atropine in controlling myopia progression shows concentration dependence [54], and 0.05% atropine is considered the most effective in controlling the equivalent spherical lens degree and axis length. However, the use of high concentrations of atropine is associated with side effects such as photophobia and blurred vision [55]. Therefore, several clinical trials have been conducted to observe the effect of 0.01% atropine on myopia control. It was found that low concentrations of atropine were effective in delaying myopia progression and slowing the growth rate of the axis length in children. Also, it reduces adverse reactions after administration and rebound reactions after discontinuation of the drug and improves compliance with atropine treatment [56, 57]. Clinical studies have also shown that orthokeratology combined with atropine is more effective than orthokeratology alone in controlling the elongation of the axis length and myopia development [58–62]. In this case, we initiated orthokeratology combined with atropine in the fourth year after surgery to better control myopia development. During the combined treatment period, low-prescription frames were worn during the day to maintain clear vision and achieve better myopia control. According to the Public Health Ophthalmology Branch of the Chinese Society of Preventive Medicine, the average total growth length of the axis length in Chinese school-age children between 6 and 15 years old is 0.93 mm [35]. The trend of the large and rapid growth of the axis length between 6 and 11 years old is reflected in the increase of the axis length in both eyes, which is 0.02 mm/year and 0.03 mm/year in the first year of treatment and 0.05 mm/year and 0.07 mm/year in the 2nd year.

In the 3th and 4th years, there was a decrease in the results of the axis length examination compared to those of the previous years. The reason for this was that the patient could not visit the hospital for a review because of the COVID-19. Additionally, the instrument used to measure the axis length was different at this time than from the previous one, which could have caused the discrepancy. Therefore, the increase in the axis length in the fourth year was 0.14 mm/year and 0.03 mm/year, indicating effective control. In this case study, in addition to keratomileusis, atropine was used to control myopic defocus and effectively improve choroidal thickness and retrobulbar perfusion, thereby controlling the rate of increase in axis length and development [45, 63, 64]. The vision was not fully corrected due to the high refractive error and flat keratometry (-5.00/-0.75 × 180 diopters in the right eye and − 6.50/-0.50 × 60 diopters in the left eye, flat keratometry/steep keratometry 41.00/43.00 in the right eye and 42.50/42.75 in the left eye). In order not to affect the patient’s quality of life and pursue better myopia control effect during the daytime, we recommended the patient to wear glasses to correct the remaining refractive error.

In conclusion, early intervention is crucial for preventing the development of congenital myopia. Particularly, the presence of posterior scleral staphyloma in this patient underscores the importance of preventing and treating myopia to avoid the progression of pathological myopia, which can lead to irreversible visual impairment. Several synthetic materials have recently been developed for posterior scleral reinforcement, such as artificial pericardial patches, polyester fiber mesh, and modified liquid silicone [9, 65]. Scleral collagen crosslinking [66] and scleral regeneration therapy [67] are potential treatments. This case involved early interventional treatment of a patient with congenital myopia using a combination of posterior scleral consolidation, optical correction with medication, and visual function training to intervene in myopia development, with a follow-up period of up to 8 years. The combined effect of posterior scleral consolidation, orthokeratology, and low concentration of atropine exhibited remarkable results in controlling myopia development, resulting in a slow increase in axial length, even below the physiological growth rate. The patient achieved stable myopic refractive error and could live and study normally, significantly improving self-confidence. This case provides a novel approach to managing congenital myopia in children and confirms that combined interventions can restore clear vision and normal visual function in patients with congenital myopia.

## Data Availability

All data generated or analyzed during this study are included in this published article.
